# Pubertal timing in children with Silver Russell syndrome compared to those born small for gestational age

**DOI:** 10.3389/fendo.2022.975511

**Published:** 2022-08-24

**Authors:** Giuseppa Patti, Federica Malerba, Maria Grazia Calevo, Maurizio Schiavone, Marco Scaglione, Emilio Casalini, Silvia Russo, Daniela Fava, Marta Bassi, Flavia Napoli, Anna Elsa Maria Allegri, Giuseppe D’Annunzio, Roberto Gastaldi, Mohamad Maghnie, Natascia Di Iorgi

**Affiliations:** ^1^ Department of Pediatrics, IRCCS Istituto Giannina Gaslini, Genova, Italy; ^2^ Department of Neuroscience, Rehabilitation, Ophthalmology, Genetics, Maternal and Child Health - University of Genova, Genova, Italy; ^3^ Epidemiology and Biostatistics Unit, Scientific Direction, IRCCS Istituto Giannina Gaslini, Genova, Italy; ^4^ Department of Pediatrics, Ospedale Ss. Annunziata, Taranto, Italy; ^5^ Cytogenetic and Molecular Genetics Laboratory, IRCCS, Istituto Auxologico Italiano, Milano, Italy

**Keywords:** puberty, bone age, silver russell syndrome, 11p15 LOM, mUPD7

## Abstract

**Context:**

Data on pubertal timing in Silver Russell syndrome (SRS) are limited.

**Design and methods:**

Retrospective observational study including twenty-three SRS patients [11p15 loss of methylation, (11p15 LOM, n=10) and maternal uniparental disomy of chromosome 7 (mUPD7, n=13)] and 21 small for gestational age (SGA). Clinical (thelarche in females; testis volume ≥ 4 ml in males; pubarche), BMI SD trend from the age of 5 to 9 years to the time of puberty, biochemical parameters of puberty onset [Luteinizing hormone (LH), 17-β-estradiol, testosterone], and bone age progression were evaluated

**Results:**

Pubertal onset and pubarche occurred significantly earlier in children with SRS than in SGA (p 0.03 and p 0.001, respectively) and clinical signs of puberty onset occurred earlier in mUPD7 than in 11p15LOM group (p 0.003). Five SRS children experienced central precocious puberty and LH, 17-β-estradiol, testosterone were detected earlier in SRS than in SGA (p 0.01; p 0.0001). Bone age delay in SRS children was followed by rapid advancement; the delta between bone age and chronological age in SRS group became significantly higher than in SGA group at the age of 9-11 years (p 0.007). 11p15LOM patients were underweight at the age of 5 years and showed a progressive normalization of BMI that was significantly higher than in mUPD7 (p 0.04) and SGA groups (p 0.03) at puberty onset.

**Conclusion:**

Timing of puberty is affected in SRS and occurred earlier in mUPD7 compared to 11p15LOM. The impact of early puberty on adult height and metabolic status deserves long-term evaluation.

## Introduction

Silver-Russell syndrome (SRS) is an epigenetic disorder characterized by severe intrauterine (IUGR) and postnatal growth retardation with typical dysmorphic features and has an incidence between 1 in 70.000 and 1 in 100.000 live births (1). The most common reported genetic abnormalities are 11p15 ICR1 loss of methylation, (11p15 LOM) and maternal uniparental disomy of chromosome 7 (mUPD7), which occur in 30-60% and 5-10% of cases, respectively (2); single cases carry other chromosome abnormalities (3–5), including duplications of maternal 11p15 (6, 7). In a significant proportion of patients (40%), the molecular etiology remains unknown, and SRS remains primarily a clinical diagnosis, according to the Netchine-Harbison (NH) scoring system and the international consensus statement (2, 8). Indeed, a target next-generation sequencing approach in patients referred for SRS testing increases the mutation rate as well as other diagnoses overlapping SRS (4, 9–11)

Data on the natural history of puberty and bone age progression in patients with SRS are limited.

It is known that children who are born small for gestational age (SGA), including those with SRS, tend to have earlier and rapidly progressing puberty, with faster bone maturation and a shorter period of pubertal peak height velocity, associated with metabolic abnormalities such as visceral adiposity (12–15). According to the available literature, onset of puberty in SRS is usually within the normal range (8–13 years in girls and 9–14 years in boys) but at the younger end of the spectrum (2, 12, 15, 16). SRS children (particularly those with 11p15LOM) can experience an early and rapidly progressive adrenarche in comparison with non-SRS SGA children (2, 12, 16, 17). In a retrospective study including 62 subjects with clinical diagnosis of SRS, Binder et al. showed that premature adrenarche was more frequent in SRS than in general population and was associated with early age at initiation of GH treatment. However, growth response to GH treatment and adult height were not compromised by early adrenarche in this cohort (17).

In SRS patients with early adrenarche, the onset of central puberty might be earlier and the tempo faster than expected. In the past few decades, population studies analyzing the timing of normal puberty observed a mean age of puberty onset of 9.7–10 years in healthy girls (2, 15), with an earlier thelarche in girls born more recently (mean age 9.86 years in European girls born in 2006 versus 10.88 years in those born in 1991) (18). One hypothesis to explain the trend towards younger age of pubertal onset in girls is that an earlier maturation can be the expression of an adaptative mechanism to escape from ectopic adiposity which, in turn, ensues from a mismatch between reduced prenatal weight gain and increased postnatal weight gain (19, 20).

SRS girls seem to start activation of hypothalamic-pituitary-gonadal axis at a mean age of 9.1 years (2) This early puberty further accelerates bone age maturation, which leads to an attenuated pubertal growth spurt and compromised adult height (2). Children with mUPD7 are likely to progress to central puberty at an even younger age than patients with SRS and 11p15 LOM (mean starting age 8.5 years in girls and 9.5 years in boys) (2). A rapid increase in body mass index (BMI) might also exacerbate the tendency to early adrenarche and central puberty (2, 15, 16).

In this study, we investigated the clinical and biochemical features of puberty onset and the evolution of bone age and BMI over time in a cohort of genetically confirmed SRS patients compared to subjects born SGA.

## Patients and methods

This is a retrospective observational study, including patients with a confirmed diagnosis of SRS recruited at the Pediatric Endocrine Unit, Istituto Giannina Gaslini, University of Genova (Genova, Italy), between November 2014 and October 2021

Population includes:

- Patients with molecular diagnosis of SRS and with an age ≥ 5 years.
- Children born SGA who were 5 years old or older at the time of enrollment; SGA subjects were rigorously selected in order to exclude chromosomopathies and syndromes; dysmorphic features, psychomotor delay, macrocephaly, microcephaly and comorbidities were exclusion criteria and a normal karyotype was mandatory.

The study was approved by the Institutional Review Board and it was approved by the ethical committee of Giannina Gaslini Institute (PRIN 2015. Number: 2015JHLY35). Written informed consent was obtained from the parents or legal guardians of all subjects according to the Declaration of Helsinki.

### Patients

The study group comprised 23 SRS subjects (11p15LOM n=10; mUPD7 n=13; 12 males; 11 females) and 21 SGA subjects (14 males; 7 females). Five SRS (3 11p15LOM; 2 mUPD7) patients (22%) required nasogastric tube feeding for the first weeks of life. Growth hormone (GH) treatment was undertaken in 18 of 23 (78%) SRS children and in 13 of 21 (61%) SGA children and the mean duration of GH treatment was 6.9 ± 3 years in SRS and 6.9± 2.9 years in SGA; the age at the start of treatment was 4.7± 2.6 years in SRS and 7.8 ± 2.6 years in SGA; the GH dose was between 0.034 and 0.035 mg/kg/day in both groups. 3 subjects with SRS and 4 with SGA reached the adult height.

### Genetic analysis

Genomic DNA was extracted from peripheral white blood cells using the Wizard^®^ Genomic DNA Purification Kit (Promega). Patients analyzed by molecular testing before 2008: from parents to proband segregation by microsatellite analysis spanning the whole chromosome 7 was performed to disclose mUPD7. Patients addressed to molecular testing after 2008: hypomethylation at H19/IGF2: IG-DMR was first analyzed by Southern-Blot hybridization of H19-DMR or by MS-MLPA kit ME030-C3 BWS/RSS (MRC-Holland, Amsterdam, The Netherlands). Patients with a balanced methylation pattern at H19/IGF2: IG-DMR underwent to mUPD7 analysis.

### H19/IGF2: IG-DMR methylation analysis

Genomic DNA was digested with Csp6I/HpaII and BamHI/NotI restriction enzymes, respectively, before southern blot hybridization to H19-DMR (provided by Prof. A. Riccio, CNR Institute of Genetics and Biophysics, Naples). MS-MLPA analysis was carried on cases and control samples according to the kit instructions and analyzed by Coffalyser.net software. The protocols and quantitative analysis to detect low level of mosaicism and aberrant copy number were previously described

### mUPD7 Analysis

A standard panel of microsatellite markers D7S517 (7p22.2), D7S513 (7p21.3), D7S507(7p21.1), D7S503 (7p21.1), D7S2493 (7p15.3), D7S2525 (7p15.2),D7S2496 (7p14.3), D7S519 (7p13), D7S2422 (7p12.1), D7S2467(7p12.1), D7S506(7p12.1), D7S1870 (7q11.23), D7S669 (7q21.1), D7S486 (7q31.2), D7S640(7q32.3), D7S798 (7q36.2), D7S2465(7q36.3) was investigated. In case of not informative markers, additional closely mapped microsatellites were analysed. PCR fragments were separated by capillary electrophoresis on the automated ABI 310 sequencer and data analysed using the Genemapper software (Applied Biosystem).

### Auxological and pubertal data

Data on gestational age as well as birth weight, birth length and head circumference were obtained from birth charts and converted to SD according to Bertino et al. (21).

Auxological data were evaluated according to Tanner growth charts (22). BMI was calculated according to Tanner growth charts and the trend was evaluated at different ages (from the age of 5 to the age of 9 years) and at the age of clinical puberty onset. In addition, weight, length and BMI data as well as weight gain from birth to 4 years and delta BMI gain from age 2 to 4 years (with a variability of ± 2 months) were collected from growth charts when available. Clinical pubertal onset was defined as testicular volume ≥ 4 ml in boys and breast development (B2 Tanner stage) in females (23).

Bone age was assessed by Greulich and Pyle method (24) and the bone age progression was evaluated from the age of 5 to the age of 11 years by the same radiologist and by the same pediatric endocrinologist (GP). Hormonal parameters [Luteinizing hormone (LH), 17-β-estradiol, testosterone] were evaluated by chemiluminescent assay (Roche). LH, 17-β-estradiol and testosterone were considered detectable if ≥0.1 U/L, ≥ 5 pg/ml, ≥5 ng/dl, respectively.

### Statistical analysis

Data are described as mean and standard deviation (SD) or median and range for continuous variables, and as absolute and relative frequencies for categorical variables.

Non parametric analysis (Mann-Whitney U-test), for continuous variables and the Chi square or Fisher’s exact test for categorical variables were used to measure differences between groups. Statistical analysis was performed using SPSS for Windows (SPSS Inc, Chicago, Illinois USA). Pairwise correlation analysis between anthropometrics data at birth, early feeding characteristics, BMI trend over time and clinical and biochemical pubertal onset data was performed; p values ≤ 0.05 were considered statistically significant, and all p values were based on two-tailed tests.

## Results

### Patient’s characteristics

The mean age at the last evaluation was 11.3 ± 3.9 SD years in SRS group and 14.2 ± 1.9 SD years in SGA group.

**Birth**. Two subjects with 11p15LOM were born by *in vitro* fertilization and Intra-Cytoplasmic Sperm Injection, respectively. The mean gestational age was 36.4 ± 2.3 weeks in SRS and 39.2± 1.5 weeks in SGA.

All 11p15LOM patients were born SGA versus 76% in mUPD7 group. Birth length SD was shorter in 11p15LOM group than in mUPD7 group (p 0.06) and SGA group (p 0.05) (**Table 1**). Birth weight (grams) was lower in SRS than in SGA (p 0.0001), although there was no significant difference in terms of birth weight SD; head circumference SD was greater in SRS than in SGA (p 0.02). Birth data and auxological data are reported in **Table 1**.

**Table 1:** Birth data and auxological data at last evaluation of SGA and SRS (divided into 11p15LOM and UPD7 groups) patients.

					p-values			
	SRS (23 pt)	11p15LOM (10 pt)	UPD7 (13 pt)	SGA (21 pt)	SRS vs SGA	11p15 vs UPD7	11p15LOM vs SGA	UPD7 vs SGA
**Gestational age (w)**	36.4 ± 2.3	36.64 ± 2.57	36.31 ± 2.10	39.2 ± 1.5	**0.0001**	0.98	**0.002**	**0.0001**
**Birth weight (gr)**	1823 ± 468	1697 ± 501	1919 ± 436	2415 ± 338	**0.0001**	0.28	**0.001**	**0.001**
**Birth weight SD**	-2.29 ± 0.75	-2.61 ± 0.75	-2.04 ± 0.67	-2.19 ± 0.42	0.93	0.06	0.12	0.25
**Birth HC (cm)**	31.93 ± 1.96	32.51 ± 2.13	31.50 ± 1.82	32.69 ± 1.28	0.48	0.13	0.80	0.18
**Birth HC SD**	-0.78 ± 0.83	-0.35 ± 0.77	-1.08 ± 0.76	-1.42 ± 0.44	**0.02**	0.11	**0.002**	0.21
**Birth length**	41.54 ± 3.71	40.50 ± 3.57	42.42 ± 3.75	45.92 ± 1.99	**0.001**	0.28	**0.001**	**0.02**
**Birth length SD**	-2.54 ± 1.06	-3.01 ± 0.98	-2.15 ± 0.99	-2.18 ± 0.77	0.34	0.06	**0.05**	0.82
**Age at last evaluation (y)**	11.3 ± 3.9	12.76 ± 4.50	10.12 ± 3.11	14.2 ± 1.9	**0.002**	0.17	0.27	**0.0001**
**Target height SD**	0.03 ± 0.80	0.10 ± 1.06	-0.02 ± 0.61	-0.76 ± 1.18	0.06	0.79	0.15	0.11
**Δ from target height SD**	-1.43 ± 1.50	-1.90 ± 1.79	-1.10 ± 1.24	-1.07 ± 1.15	0.53	0.43	0.35	0.89

w, weeks; y, years; SGA, small for gestational age; SRS, Silver-Russell syndrome; Δ, delta; gr, grams; HC, head circumference; 11p15 LOM, 11p15 loss of methylation; UPD7, maternal uniparental disomy of chromosome 7.The bold numbers indicate a statically significant p-value ( p value ≤ 0.05).

**Pubertal onset.** The mean age at the last evaluation was 11.3 ± 3.9 SD years in SRS group and 14.2 ± 1.9 SD years in SGA group. Clinical pubertal onset and pubarche occurred significantly earlier in SRS group than in SGA group (p 0.03 and p 0.001, respectively) and serum LH and sexual hormones became detectable earlier in SRS than in SGA (p 0.01; p 0.0001), (**Table 2**).

**Table 2:** Puberty onset characteristics in SRS and SGA groups.

					p-values			
	SRS	11p15LOM	UPD7	SGA	SRS vs SGA	11p15 vs UPD7	11p15 vs SGA	UPD7 vs SGA
**Clinical puberty onset (y)**	10.02 ± 1.80 (13 pt) [7.0-13.0]	11.75 ± 1.14 (5 pt) [10.0-13.0]	8.94 ± 1.15 (8 pt) [7.0-10.0]	11.38 ± 1.15 (18 pt) [8.0-13.5]	**0.03**	**0.003**	0.53	**0.0001**
**Clinical puberty onset (y) in males**	11.46 ± 1.25 (6 pt) [8.0-13.0]	12.13 (4 pt) [11.5-13.0]	10.0 (2 pt) [10.0]	11.79 ± 1.48 (12 pt) [8.0-13.5]	0.61	**-**	–	**-**
**Clinical puberty onset (y) in females**	8.79 ± 1.15 (7 pt) [7.0-10.0]	10.0 (1 pt)	9.0 (6 pt) [7.0-10.0]	10.57 ± 0.96 (6 pt) [9.25-12.0]	**0.02**	**-**	–	**-**
**Pubarche age (y)**	9.92 ± 1.38 (14 pt) [7.5-11.5]	10.34 ± 1.66 (7 pt) [7.5-11.5]	9.49 ± 0.97 (7 pt) [8.0-10.5]	11.90 ± 1.70 (18 pt) [8.0-14.5]	**0.001**	0.16	**0.04**	**0.002**
**Pubarche age (y) in males**	10.33 ± 1.49 (8 pt) [7.5-11.5]	11.5 (5 pt) [7.5-11.5]	10.0 (3 pt) [8.8-10.4]	12.37 ± 1.80 (12 pt) [8.0-14.5]	**0.01**	–	**-**	**-**
**Pubarche age (y) in females**	9.37 ± 1.10 (6 pt) [8.0-10.5]	9.50 (2 pt) [8.5-10.5]	9.38 (4 pt) [8.0-10.5]	10.97 ± 1.06 (6 pt) [9.8-12.5]	0.09	–	**-**	**-**
Measurable sexual hormones*, age (y)	9.47 ± 1.44 (12 pt) [7.0-12.3]	10.12 ± 1.61 (5 pt) [8.0-12.3]	9.01 ± 1.22 (7 pt) [7.0-10.8]	11.86 ± 1.28 (19 pt) [9.8-14.7]	**0.0001**	0.27	**0.02**	**0.0001**
Measurable sexual hormones*, age (y) in males	9.92 ± 1.36 (8 pt) [8.0-12.3]	9.82 (5 pt) [8.0-12.3]	9.19 (3 pt) [8.8-10.8]	12.09 ± 1.39 (13 pt) [9.9-14.7]	**0.01**	–	**-**	**-**
Measurable sexual hormones*, age (y) in females	8.58 ± 1.30 (4 pt) [7.0-10.1]	0 pt	8.59 (4 pt) [7.0-10.1]	11.38 ± 0.95 (6 pt) [9.8-12.7]	**0.03**	–	**-**	**-**
Measurable LH**, age (y)	9.01 ± 1.53 (13 pt) [6.0-11.3]	9.58 ± 2.07 (4 pt) [6.6-11.3]	8.75 ± 1.29 (9 pt) [6.0-10.1]	10.67 ± 1.75 (16 pt) [7.4-14.0]	**0.01**	0.20	0.49	**0.005**
Measurable LH**, age (y) in males	9.47 ± 1.50 (7 pt) [6.6-11.3]	10.04 (4 pt) [6.6-11.3]	9.08 (3 pt) [8.8-9.8]	10.9 ± 1.72 (12 pt) [8.0-14]	0.1	–	–	**-**
Measurable LH**, age (y) in females	8.47 ± 1.51 (6 pt) [6.0-10.1]	0 pt	8.01 (6 pt) [6.0-10.1]	9.99 ± 1.90 (4 pt) [7.4-11.9]	0.3	–	–	**-**
**Menarche age (y)**	11.11 ± 0.69 (5 pt) [10.0-11.8]	10.91 ± 1.29 (2 pt) [10.0-11.8]	11.23 ± 0.25 (3 pt) [11.0-11.5]	11.86 ± 0.75 (4 pt) [10.9-12.7]	0.29	1	0.53	0.40

y, years; SRS, Silver-Russell syndrome; 11p15, 11p15 loss of methylation; UPD7, maternal uniparental disomy of chromosome 7; pt, patients; LH, luteinizing hormone * 17- β -estradiol ≥ 5 pg/ml; testosterone ≥ 5 ng/dl. ** LH ≥ 0.1 U/L.

**-** not applicable because of the little number of patients.The bold numbers indicate a statically significant p-value ( p value ≤ 0.05).

mUPD7 patients presented a significantly earlier pubertal onset compared to11p15LOM subjects (p 0.003). The age of pubarche as well as the age at which sexual hormones became detectable in serum was earlier in mUPD7 if compared to 11p15 LOM group, although not significantly (p 0.16 and p 0.27, respectively) (**Table 2**). Five SRS children, 21.7% of the group, (3 females and 2 males, 2 11p15LOM and 3 mUPD7) experienced central precocious puberty; 4 out of 5 were treated with gonadotropin-releasing hormone (GnRH) analog; 1 girl who came to our attention at the age of 15 years had experienced menarche at the age of 10 years while the two boys (1 with 11p15LOM; 1 with mUPD7) showed a biochemical central precocious pubertal activation but with small testis (testicular volume 3 ml in both subjects).

**Figure 1:**
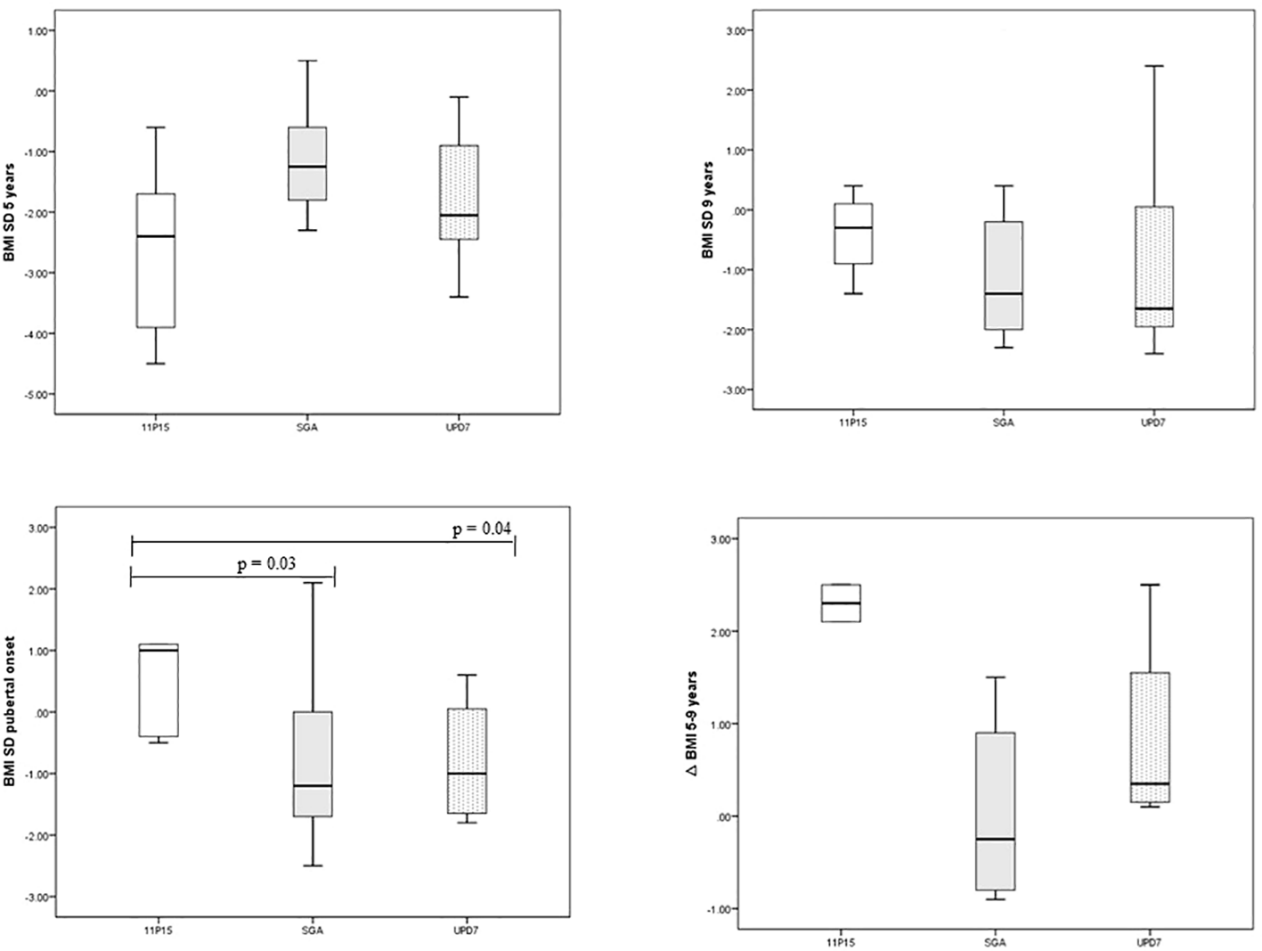
BMI trend in SRS and in SGA subjects.

**BMI evolution and feeding problems.** Patients with 11p15LOM were underweight at the age of 5 years, then showed a progressive normalization of BMI over time and a significantly higher BMI SD at the age of puberty onset compared to mUPD7 group (p 0.04) and SGA group (p 0.03) (**Table 3**), (**Figure 1**).

**Table 3:** BMI trend between 5 to 9 years related to bone age in SGA and SRS patients.

					p-values			
	SRS (23 pt)	11p15LOM (10 pt)	UPD7 (13 pt)	SGA (21 pt)	SRS vs SGA	11p15 vs UPD7	11p15LOM vs SGA	UPD7 vs SGA
**BMI SD at 5y**	-2.07 ± 1.16	-2.58 ± 1.43	-1.80 ± 0.97	-1.12 ± 0.98	0.90	0.33	0.09	0.18
**BMI SD at 7y**	-1.48 ± 1.30	-1.63 ± 1.48	-1.39 ± 1.25	-1.41 ± 1.01	0.98	0.96	1	0.98
**BMI SD at 9y**	-0.72 ± 1.39	-0.40 ± 0.75	-0.89 ± 1.64	-0.84 ± 1.46	0.85	0.28	0.35	0.70
**BMI SD at puberty onset**	-0.31 ± 1.09	0.46 ± 0.83	-0.80 ± 0.97	-0.84 ± 1.20	0.20	**0.04**	**0.03**	0.85
**Δ BMI from 5y to 9y**	1.13 ± 1.02	2.3 ± 0.28	0.84 ± 0.91	0.03 ± 0.98	**0.04**	0.09	0.07	0.11
**Δ BMI from 7y to 9y**	0.75 ± 1.04	1.47 ± 1.25	0.48 ± 0.89	0.03 ± 0.36	0.06	0.08	**0.007**	0.31
**Bone age-chronological age (at 5/7y)**	-1.71 ± 0.81	-1.32 ± 0.86	-1.92 ± 0.73	-1.55 ± 0.95	0.79	0.26	0.61	0.46
**Bone age-chronological age (at 7/9y)**	-1.03 ± 0.97	-0.33 ± 0.78	-1.37 ± 0.89	-1.73 ± 1.24	0.15	**0.05**	**0.04**	0.46
**Bone age-chronological age (at 9/11y)**	-0.18 ± 0.98	-0.57 ± 1.23	0.16 ± 0.62	-1.42 ± 1.25	**0.007**	0.14	0.38	**0.001**
**Δ BA-CA from 5/7y to 9/11y**	2.16 ± 0.86	1.7 ± 1.2	2.39 ± 0.65	0.43 ± 0.9	**0.001**	0.71	0.08	**0.001**
**Δ BA-CA from 7/9y to 9/11y**	1.38 ± 1.02	0.78 ± 0.68	1.63 ± 1.07	0.65 ± 0.52	0.08	0.38	0.78	**0.04**

BMI, body mass index; w, weeks; y, years; SGA, small for gestational age; SRS, Silver-Russell syndrome; 11p15, 11p15 loss of methylation; UPD7, maternal uniparental disomy of chromosome 7; BA, bone age; CA, chronological age; Δ, delta.The bold numbers indicate a statically significant p-value ( p value ≤ 0.05).

Similarly, the gap between bone age and chronological age (negative at the age of 5-7 years) in SRS group became significantly higher than in SGA group at the age of 9-11 years (p 0.007) (**Table 3**).

Although no significant correlation between nasogastric tube feeding and early pubertal onset was found because of the small number of patients, it should be considered that 3 out of 5 tube-fed subjects experienced precocious puberty (60%). Available data of weight and length gain from birth to 4 years (with a variability of ± 2 months) are reported in **Table 4**.

**Table 4:** Available data of weight and length gain from birth to 4 years (variability ± 2 months) in SRS children and SGA subjects.

	11p15LOM (8pt)	UPD7 (11 pt)	SGA(7pt)	p value 11p15 vs UPD7	11p15 vs SGA	UPD7 vs SGA
**BMI SD at 2y**	-2.1 ± 1.5	-3.07 ± 0.93	-1.17 ± 0.54	0.34	0.34	**0.001**
**Δ BMI SD from 2y to 3y**	-1.45 ± 2.7	0.53 ± 0.64		**0.04**		
**Δ BMI SD from 2y to 4y**	-1.3 ± 3	0.7 ± 0.6		0.06		
**Weight gain (kg) birth-1 year**	4.1 ± 0.93	3.3 ± 0.56		0.23		
**Weight gain (kg)** **Birth-4 years**	8.63 ± 1.3	8.1 ± 1.44	9.6 ± 1.07	0.33	0.16	**0.03**
**Weight gain (kg)** **2-4 years**	2.5 ± 1.6	3.1 ± 1.1		0.66		
**Length gain** **Birth-1 year**	23 ± 8.2	22.1 ± 4.5		0.5		
**Height gain birth-4years**	49.5 ± 6.6	44.9 ± 4.9	47.3 ± 4.2	0.07	0.34	0.37
**Height gain (cm) 2-4 years**	16.7 ± 3.6	15 ± 3.3		0.34		

BMI, body mass index; w, weeks; y, years; SGA, small for gestational age; SRS, Silver-Russell syndrome; 11p15, 11p15 loss of methylation; UPD7, maternal uniparental disomy of chromosome 7.The bold numbers indicate a statically significant p-value ( p value ≤ 0.05).

## Discussion

To our knowledge, this is the first study that evaluated the clinical and biochemical characteristics of pubertal timing in association with the progression of bone age and BMI trends in genetically confirmed SRS children compared to children born SGA.

The published literature on the natural history of puberty onset and bone age progression in patients with SRS is limited (2, 16, 17, 25, 26). In a retrospective study including 62 subjects with clinical diagnosis of SRS, Binder et al. showed that premature adrenarche was more frequent in SRS than in the general population and was associated with early age at initiation of GH treatment, but growth response to GH treatment and adult height were not compromised (17). The lack of a molecular diagnostic confirmation represents a significant limitation of this study preventing a comparison between molecular SRS groups (17). In 16 subjects with 11p15LOM, Canton et al. (16) showed that the age of onset of adrenarche was earlier than in general population and a marked increase in BMI was associated with premature adrenarche and early puberty. However, this study included only one molecular SRS group (11p15LOM) (16).

In a study comparing a cohort of SRS subjects (31 11p15 LOM, 11 mUPD7, 20 idiopathic SRS) and a cohort of patients born SGA non-SRS, puberty started significantly earlier in the former (at 10.2 years versus 11.2 years in girls with SRS and non-SRS SGA, respectively, and at 11.4 years versus 12.0 years in boys with SRS and non-SRS SGA, respectively). In this study, boys with mUPD7 were the youngest at the onset of puberty and in 17 SRS patients puberty was postponed for 2 years with GnRH analogs due to a low predicted adult height (25). In a study including 31 SRS patients (15 11p15LOM, 7 mUPD7, 9 clinical diagnosis) and 123 non-SRS SGA subjects, Goedegebuure et al. showed a similar onset and progression of puberty in SRS and non-SRS SGA subjects (26).

Although there is little data on puberty onset in SRS, children born SGA are known to have an increased risk of developing early and rapidly progressing puberty, compromising thereby adult height and metabolic status (12, 13, 27–29). Nevertheless, limited data are available so far on therapeutic options in SGA children, including SRS, with a poor adult height expectation, with the exception of few studies on the use of GnRH analogs in combination with GH (12, 30–33). A double-blind clinical trial is still ongoing to investigate the efficacy of Anastrozole, a third-generation aromatase inhibitor, in slowing bone maturation in SRS (34).

It should be considered that a secular trend towards an earlier puberty onset in healthy children was observed in many countries. One hypothesis to explain the trend towards younger age of pubertal onset in girls is that an earlier maturation can be the expression of an adaptative mechanism to escape from ectopic adiposity which, in turn, ensues from a mismatch between reduced prenatal weight gain and increased postnatal weight gain (19, 20). Yanhui et al. have also recently showed an association between prepubertal adiposity and earlier puberty onset both in females and in males (35).

Since most SRS subjects are born SGA, the aim of our study was to evaluate the clinical and biochemical puberty characteristics in SRS compared to non-SRS SGA subjects and to evaluate the impact of BMI on puberty onset. In our cohort, puberty onset and pubarche occurred significantly earlier in SRS group than in SGA group and LH and sexual hormones were detected earlier in SRS than in SGA. In particular, within the SRS group, clinical signs of pubertal onset occurred earlier in mUPD7 than in 11p15LOM subjects. In addition, according to the literature, our SRS group showed a delay of bone age followed by rapid acceleration at the age of 9-11 years (2).

In agreement with Canton et al. (16), we found that the BMI of SRS children progressively increases with age. Notably, patients with 11p15LOM who were underweight at the age of 5 years showed a progressive normalization of BMI which was significantly higher at the time of onset of puberty compared to BMI of mUPD7 and SGA groups. However, the observation that children with mUPD7 experienced an earlier pubertal onset than 11p15LOM despite the higher BMI increase between 5 and 9 years in 11p15LOM is in partial contrast with the findings of Canton et al. With the limitation of the fact that our cohort includes both 11p15LOM and mUPD7 subjects while Canton’s cohort includes only 11p15LOM subjects, the lack of a correlation between weight/BMI gain and the timing of puberty in our cohort is not in line with the hypothesis of the key role of a marked BMI increase as causative of early puberty in SRS (16). Although GH administration does not appear to have a negative impact on the progression of puberty (our patients are treated with similar GH dosage) and testicular volume (36) and that of GnRH analogs on increasing BMI (37), we cannot completely rule out their role in our cohort. However, it should be considered that GH treatment has never been performed in 3 out of 5 SRS subjects with precocious puberty in our cohort.

The lack of a correlation between weight and BMI gain and pubertal onset in our cohort as well as the emerging data in literature on the role of imprinted genes in pubertal timing (38, 39) may suggest that imprinted genes involved in SRS can play a role in the timing of puberty. While it is known that the time of puberty has a strong genetic component, recently epigenetics has been implicated as an important regulatory mechanism underlying not only the developmental process by which GnRH release is first kept in check before puberty, but also the increase in GnRH secretion (38). Loss of function mutations in Makorin Ring Finger Protein 3 (MKRN3), a maternally imprinted gene on chromosome 15, are identified genetic causes of central precocious puberty (39). More recently, several mutations in a second maternally imprinted gene, Delta-like noncanonical Notch ligand (DLK1) have also been associated with central precocious puberty (39). Given the role of mutations in the imprinted genes MKRN3 and DLK1 in pubertal timing, other imprinted candidate genes should be considered for a role in puberty initiation (39).

The two SRS boys with biochemical parameters of precocious puberty had small testes. This characteristics has been previously described in a study including 11 SRS boys (6 11p15 LOM and 5 idiopathic SRS) and can be the sign of an impaired gonadal function (40). Taking this finding into account, the assessment of testicular size in these children could underestimate the pubertal stage making it unreliable for the estimation of pubertal development in this group of patients. Low levels of inhibin B, indicating Sertoli cell dysfunction, have been reported by Goedegebuure et al. in 4 out of 14 post-pubertal SRS (26). Our study has strengths and limitations. The number of patients for a rare disease and the strict diagnostic criteria (clinical diagnosis according to NH scoring system confirmed by molecular analysis) represent the strengths, while the small number of sex-related molecular subtypes and the different mean age of SRS and SGA groups are limitations.

The different mean age of SRS and SGA groups can be explained by the strict diagnostic criteria as well as by the fact that while the SRS subjects are referred to our University Center since their first months/years of life, SGA subjects are often followed at the local hospitals and consequently they often come to our attention late. In conclusion, SRS children should be carefully monitored for signs of early puberty onset. It should be considered that SRS boys may have testicular hypoplasia and sex hormone assessment could be valuable. Since testicular hypoplasia can be a sign of gonadal disfunction, measurement of inhibin B and anti-mullerian hormone could be useful in SRS male adults. Understanding the pubertal characteristics in SRS can help define the best preventive measures (avoiding overfeeding by nasogastric tube, close pubertal monitoring and bone age assessments) and the appropriate time window for therapeutic intervention (aromatase inhibitors, GnRH analogs) when needed. Additional data are required to better understand the potential role of epigenetics in puberty and gonadal function as well as the impact of BMI gain on pubertal onset, adult height and long-term metabolic outcomes.

## Data availability statement

The original contributions presented in the study are included in the article/supplementary material. Further inquiries can be directed to the corresponding author.

## Author contributions

GP designed the study, examined the clinical phenotypes of the patients, took care of patients’ follow-up, collected data, drafted and revised the manuscript. FM took care of patients, collected data, drafted and revised the manuscript. MC performed the statistical analyses. MSca, MSch, EC helped in following the patients and in data collection. SR performed molecular analysis. DF, MB, FN, AA, GD’A, RG helped in following the patients. MM designed the study, drafted and revised the manuscript. NDI designed the study, drafted and revised the manuscript. All authors contributed to the article and approved the submitted version.

## Funding

Funder: Ministero Dell’Istruzione, dell’Università e della Ricerca. PRIN 2015. Number: 2015JHLY35. The research was partially funded by the Italian Ministry of Health.

## Acknowledgments

We are grateful for the Department of Neuroscience, Rehabilitation, Ophtalmology, Genetics, Maternal and Child Health (DINOGMI), University of Genova - a Department of Excellence - for the support in the several steps leading to the approval and publishing the study.

## Conflict of interest

The authors declare that the research was conducted in the absence of any commercial or financial relationships that could be construed as a potential conflict of interest.

## Publisher’s note

All claims expressed in this article are solely those of the authors and do not necessarily represent those of their affiliated organizations, or those of the publisher, the editors and the reviewers. Any product that may be evaluated in this article, or claim that may be made by its manufacturer, is not guaranteed or endorsed by the publisher.
