# Corrigendum: Pubertal timing in children with Silver Russell syndrome compared to those born small for gestational age

**DOI:** 10.3389/fendo.2023.1172736

**Published:** 2023-03-15

**Authors:** Giuseppa Patti, Federica Malerba, Maria Grazia Calevo, Maurizio Schiavone, Marco Scaglione, Emilio Casalini, Silvia Russo, Daniela Fava, Marta Bassi, Flavia Napoli, Anna Elsa Maria Allegri, Giuseppe D’Annunzio, Roberto Gastaldi, Mohamad Maghnie, Natascia Di Iorgi

**Affiliations:** ^1^ Department of Pediatrics, IRCCS Istituto Giannina Gaslini, Genova, Italy; ^2^ Department of Neuroscience, Rehabilitation, Ophthalmology, Genetics, Maternal and Child Health - University of Genova, Genova, Italy; ^3^ Epidemiology and Biostatistics Unit, Scientific Direction, IRCCS Istituto Giannina Gaslini, Genova, Italy; ^4^ Department of Pediatrics, Ospedale Ss. Annunziata, Taranto, Italy; ^5^ Cytogenetic and Molecular Genetics Laboratory, IRCCS, Istituto Auxologico Italiano, Milano, Italy

**Keywords:** puberty, bone age, silver russell syndrome, 11p15 LOM, mUPD7

**Incorrect Affiliation**

In the published article, there was an error in affiliation 5. Instead of “Cytogenetic and Molecular Genetics Laboratory, Istituto Auxologico Italiano, Milano, Italy”, it should be “Cytogenetic and Molecular Genetics Laboratory, IRCCS, Istituto Auxologico Italiano, Milano, Italy”.

The authors apologize for this error and state that this does not change the scientific conclusions of the article in any way. The original article has been updated.

**Incorrect Funding**

In the published article, there was an error in the Funding statement. Mention to the funding received from the Italian Ministry of Health was missing. The correct Funding statement appears below.

**FUNDING**

Funder: Ministero Dell’Istruzione, dell’Università e della Ricerca. PRIN 2015. Number: 2015JHLY35. The research was partially funded by the Italian Ministry of Health.

The authors apologize for this error and state that this does not change the scientific conclusions of the article in any way. The original article has been updated.

